# Dynamic Strain Imaging in Hypertrophic Cardiomyopathy: Refining Risk Stratification Beyond Conventional Metrics

**DOI:** 10.31083/RCM47720

**Published:** 2026-03-09

**Authors:** Weihao Cheng

**Affiliations:** ^1^School of Computer Science and Technology, Hangzhou Dianzi University, 310018 Hangzhou, Zhejiang, China; ^2^School of Communication Engineering, Hangzhou Dianzi University, 310018 Hangzhou, Zhejiang, China

We read with great interest the original research article titled, “Exercise Stress Echocardiography Predicts Adverse Cardiovascular Events in Hypertrophic Cardiomyopathy: A 5-Year Prospective Study” by Su *et al*. [1]. This well-designed prospective study integrates exercise stress echocardiography (ESE) with left atrial strain (LAS) assessment and incorporates a rigorous five-year follow-up, offering a novel and clinically meaningful framework for risk stratification in patients with hypertrophic cardiomyopathy (HCM). By demonstrating the predictive value of ESE-derived LAS for adverse cardiovascular events (ACEs), the authors provide important evidence supporting the role of dynamic functional assessment beyond conventional resting parameters.

We especially appreciate the authors’ extension of LAS—a biomarker widely validated in heart failure with preserved ejection fraction (HFpEF) [2]—to the HCM population. This translational application broadens the clinical relevance of LAS and strengthens its potential utility in diverse forms of diastolic dysfunction and myocardial remodeling.

In the spirit of constructive and collegial discussion, we would like to offer several targeted suggestions aimed at further enhancing the methodological rigor, reproducibility, and clinical applicability of this valuable work.

First, given that speckle-tracking–derived strain measurements are sensitive to ultrasound hardware and post-processing algorithms, we suggest clarifying whether the same ultrasound equipment and strain analysis software versions were consistently used throughout the entire five-year follow-up period. Consistency in imaging platforms is particularly important in longitudinal studies to minimize measurement variability unrelated to disease progression.

Second, we recommend providing more detailed information in the Methods section regarding frame rate control during ESE acquisition. Prior methodological studies have demonstrated that frame rate substantially influences the accuracy of two-dimensional speckle-tracking strain measurements, with rates above 60 frames per second generally recommended to ensure reliable deformation analysis [3]. Explicit reporting of frame rate settings would further strengthen the reproducibility of the results and facilitate comparison with future studies.

From a clinical translation perspective, the Discussion section could be enriched by exploring a specific LAS cut-off value derived from the observed 5-year incidence of ACE within the cohort. In addition to modeling LAS as a continuous variable, identifying a clinically interpretable threshold and evaluating its additive predictive value compared with established risk stratification tools—such as the 2014 ESC HCM-Risk SCD model [4]—would enhance the practical applicability of ESE-LAS integration in routine clinical decision-making.

Finally, we suggest adding a potential direction for future research: investigating whether the change rate of LAS from rest to post-exercise provides superior prognostic information compared with a single resting or peak-stress LAS measurement. Such a dynamic parameter may better reflect left atrial myocardial reserve and capture early, subtle myocardial dysfunction associated with disease progression in HCM.

We commend the authors for this thoughtful and methodologically sound contribution to HCM risk assessment and appreciate the opportunity to participate in this constructive academic dialogue. We look forward to future studies building upon this important work.
